# Management of Biliary Complications in Liver Transplant Recipients with Duct-To-Duct Anastomosis: A Single-Center Experience

**DOI:** 10.5152/tjg.2023.22724

**Published:** 2023-02-01

**Authors:** Muhammed Fatih Karakaya, Erdem Er, Onur Kırımker, Mesut Gümüşsoy, Emin Bodakçi, Mubin Özercan, Beyza Doğanay Erdoğan, Hale Gökcan, Meltem Koloğlu, Kaan Karayalçın, Cihan Yurdaydın, Acar Tüzüner, Selçuk Haznedaroğlu, Kubilay Çınar, Hasan Özkan, Ramazan Idilman

**Affiliations:** 1 Department of Gastroenterology, Ankara University Faculty of Medicine, Ankara, Turkey; 2 Department of General Surgery, Ankara University Faculty of Medicine, Ankara, Turkey; 3 Department of Biostatistics, Ankara University Faculty of Medicine, Ankara, Turkey

**Keywords:** Biliary complications, biliary leakage, biliary stricture, liver transplantation

## Abstract

**Background:**

The aims of this study were to investigate biliary complications in liver transplant recipients with choledochocholedochostomy anastomosis, to identify the risk factors for the development of such complications, and to evaluate the success of endoscopic approaches in liver transplant recipients.

**Methods:**

Between January 2013 and May 2021, a total of 238 patients with liver diseases underwent liver transplantation: 174 recipients undergoing choledochocholedochostomy anastomosis were included in the analysis.

**Results:**

Their median age was 54.0 years. The median posttransplant follow-up period was 29 months. Hepatitis B virus infection (33%) was the most common indication for liver transplantation. Most patients (87%) received living donor liver transplantation. The overall prevalence of posttransplant biliary complications was 31%. Anastomotic biliary strictures were the most common biliary complications (72%), followed by biliary leakage (13%). The median time between endoscopic retrograde cholangiography and liver transplantation was 4 months, with a mean of 3 ± 1.6 sessions. Endoscopic retrograde cholangiography-guided drainage and balloon dilation with or without stent placement was the most common treatment modalities for recipients with biliary strictures. The overall success rate of endoscopic treatment modalities was 83.3%, with 65% of the recipients exhibiting complete biochemical and endoscopic responses. The response did not differ significantly between living donor liver transplantation and cadaveric donor liver transplant recipients (*P* > .05). Three recipients required revision surgery for biliary complication repair. Six patients died due to biliary sepsis.

**Conclusion:**

Biliary stricture and leakages were the most common biliary complications after liver transplantation. Endoscopic treatment was successful in most recipients.

**Main Points**

- Posttransplantation biliary complications are an important cause of morbidity and mortality after liver transplantation.
- Biliary stenosis and biliary leakage are the most common posttransplant biliary complications.
- Endoscopic retrograde cholangiography is the first-line treatment choice for biliary complications following liver transplantation.

## Introduction

Liver transplantation (LT) is a curative treatment approach for acute, chronic end-stage liver disease and hepatocellular cancer (HCC).^1^ Posttransplant biliary complications remain a major problem, with high morbidity and mortality rates.^2-4^ The rates of biliary complications following LT range from approximately 10% to 15% in deceased donor liver transplant recipients and from 15% to 30% in living donor (LD) liver transplant recipients.^2-4^ Biliary stenosis, biliary leakage, non-anastomotic strictures, and stones are the most common posttransplant complications.^2,5,6^ More than half of all biliary complications occur at the anastomotic site during the early posttransplant period.^2,5,6^

Several factors including recipient-related factors (e.g., primary sclerosing cholangitis), graft-related issues (e.g., split grafts), surgical technique factors (type of biliary reconstruction), concomitant vascular complications, and infection are associated with biliary complications.^7-10^ However, the data are often conflicting. The aims of this study were to investigate biliary complications in liver transplant recipients undergoing choledochocholedochostomy (CDCD) anastomosis at a single center, to identify the risk factors for the development of such complications, and to evaluate the success of endoscopic approaches in these recipients.

## Materials and Methods

### Patients

Between January 2013 and May 2021, adult liver transplant recipients followed up at the Liver Disease Outpatient Clinic of the Department of Gastroenterology of Ankara University Faculty of Medicine were retrospectively evaluated. Recipients undergoing Roux-en-Y (R-Y) hepaticojejunostomy, recipients who died within the first month of LT, and recipients who were lost to follow-up were excluded. Patients with hepatic artery thrombosis, primary nonfunction, and graft rejection were also excluded. Data were collected from the outpatient visit charts. The present study was established in accordance with Helsinki Declaration and was approved by the Ethics Committee of the Ankara University Faculty of Medicine (No. 2021/267; August 4, 2021).

### Immunosuppression

The immunosuppressive protocol consisted of tacrolimus or cyclosporine plus mycophenolate mofetil and a steroid. Tacrolimus or cyclosporine was administered at a therapeutic target level. Corticosteroids were tapered over 12 weeks and discontinued 24-48 weeks after LT if necessary. Alternative immunosuppressive agents, including sirolimus or everolimus, were used in some patients who were intolerant of calcineurin inhibitors.

Biliary complications were identified based on the clinical signs of fever, jaundice and/or right upper quadrant abdominal pain, biochemical findings of abnormal serum aminotransferases, bilirubin, alkaline phosphatase, gamma glutamyl transferase levels, and radiological studies, including abdominal ultrasonography (US), magnetic resonance cholangiography (MRC), endoscopic retrograde cholangiography (ERC), and percutaneous transhepatic cholangiography (PTC). An anastomotic biliary stricture was defined as segmental narrowing around the biliary anastomosis site. Biliary leakage was defined as bile leakage into the abdomen.^7,8^

The management of biliary complications was divided into 2 approaches, ERC-guided and PTC-guided drainages, as previously described.^11^ A complete response was defined as a normal biochemical test and no biliary strictures and/or leakage on abdominal US, MRC, or ERC.

All liver transplant recipients were seen at regular intervals at the Liver Disease Outpatient Clinic. A physical examination and biochemical, serological, and virological tests were performed at each visit.

### Statistical Analysis

In descriptive statistics, continuous variables were expressed as means ± standard deviations or medians and ranges, and categorical variables were expressed as frequencies and percentages. Age, body mass index, albumin, creatinine, and Model for End-Stage Liver Disease (MELD) scores with a normal distribution were compared between patients with and without biliary complications using Student’s *t*-test or the Mann–Whitney *U* test. The chi-squared test was used to evaluate the differences between the 2 groups in terms of gender, emergency transplant status, graft type, and HCC status. Multivariate logistic regression analysis was performed to evaluate the risk factors for biliary complications, including recipients and donor characteristics and perioperative parameters. Values of *P* < .05 were considered statistically significant. The Statistical Package for the Social Sciences version 15 (SPSS Inc.; Chicago, IL, USA) was used for the statistical analysis.

## Results

A total of 238 patients with liver diseases underwent LT during the study period. Among them, a total of 174 patients undergoing CDCD anastomosis met the inclusion criteria. The patients were predominantly male (64.9%), and their mean age was 51.2 ± 12.2 years (median age: 54.0 years). The median follow-up time was 29 months (range: 1-106 months). Chronic viral hepatitis was the most common indication for LT (44%), including hepatitis B virus (HBV)-induced cirrhosis (n = 57; 33%), hepatitis C virus (HCV)-induced cirrhosis (n = 10; 5.7%), and hepatitis D virus (HDV)-induced cirrhosis (n = 10; 5.7%), followed by cryptogenic cirrhosis (n = 34; 19.5%), autoimmune liver diseases (n = 20; 11.4%), alcohol-related liver disease (ALD) (n = 13; 7.5%), Wilson’s disease (n = 7; 4%), non-alcoholic fatty liver disease-related cirrhosis (n = 6; 3.4%), and miscellaneous diseases (n = 6). Hepatocellular cancer was detected in 38 (23.3%) cirrhotic patients. Liver transplantation was performed on 11 (6.3%) patients due to acute liver failure (3 patients with HBV-related, 5 with toxic hepatitis, 2 with autoimmune hepatitis, and 1 with Budd-Chiari syndrome). Around 151 (87%) patients received LDLTs, while 23 (13%) patients received cadaveric donor LT. Most patients were on tacrolimus-based triple combination therapy (tacrolimus and prednisolone plus MMF) or cyclosporine-based therapy, and 29.8% sirolimus or everolimus-based therapy.

The rate of biliary complications was 31% (n = 54). Most (64.8%) patients with biliary complications were male, and their mean age was 51.8 ± 12.0 years. Anastomotic biliary strictures were the most common biliary complications (n = 39; 72.2%), followed by biliary leakage (n = 7; 13%) and biliary strictures with biliary leakage (n = 7; 13%). Biliary stones were present in 25% of the patients with anastomotic biliary stenosis, whereas isolated choledocholithiasis was observed in 1 patient (Table 1).

Endoscopic retrograde cholangiography was performed on all patients with biliary complications. The median time between ERC and LT was 4 months (range: 1-36 months). About 61% of these recipients underwent ERC within the first 6 months after LT. Endoscopic retrograde cholangiography procedures required a median of 3 sessions (range: 1-8 sessions). Patients undergoing ERC were hospitalized for 1 or 2 days after the procedure and followed using a conservative strategy. No major ERC-related complications were observed. Mild abdominal discomfort, transient asymptomatic hyperamylasemia, and mild to moderate pancreatitis were observed in some cases.

Endoscopic retrograde cholangiography-guided drainage and balloon dilatation with or without stent placement were the most common treatment modalities in patients with biliary strictures (Figure 1A, B, and C), whereas ERC-guided drainage and stent placement were the most common treatment modalities in patients with biliary leakage (Table 2) (Figure 2A, B, C, and D). The overall success rate of endoscopic treatment modalities was 83.3% (n = 45), with 65% (n = 35) of the recipients exhibiting complete biochemical and endoscopic responses. The success rate did not differ significantly between the LDLT and cadaveric donor LT recipients (83.3% vs. 83.3%, *P* > .05). Three LDLT recipients with anastomotic strictures (5.6%) required revision surgery. Roux-en-Y reconstruction was the preferred type of surgical biliary reconstruction for salvage therapy. Six patients were successfully treated using the percutaneous rendezvous method and magnet (Table 2).

Logistic regression analysis revealed no significant association between any variable and biliary complications (Table 3).

A total of 6 recipients died within a median of 3 months (range: 1-7 months) after LT due to biliary infection and sepsis. Among them, 3 had anastomotic stenosis, and the remaining 3 recipients had biliary leakage.

## Discussion

In this study, we investigated biliary complications in liver transplant recipients undergoing CDCD anastomosis and identified the risk factors for the development of biliary complications. Hepatitis B virus-induced liver disease was the most common indication for LT. The prevalence of biliary complications following LT was 31%: anastomotic biliary structures (39 cases/174 recipients), biliary leakage (7 cases/174 recipients), and biliary stricture and leakage (7 cases/174 recipients) were the most common biliary complications. Most complications (61%) were seen in the early posttransplant period. These results are consistent with previous reports.^2, 5-7, 11-13^ A systematic review of 61 studies found that the overall biliary stricture and leakage rates were 13% (1844 cases/14 359 recipients) and 8% (936 cases/11 397 recipients), respectively.^5^ A UK registry analysis reported biliary complication rates of 9%: biliary stricture was 4%, biliary leakage 4%, and biliary stricture and leakage 1.4%.^2^ A recent study in Turkey documented biliary complications in recipients, who underwent ERC between September 2005 and January 2015. Endoscopic retrograde cholangiography was performed in 446 cases of 1136 recipients with LDLT. The investigators reported anastomotic biliary structures and biliary leakage were the most common biliary complications.^13^ These results indicate that biliary anastomotic strictures and biliary leakages are the most common biliary complications following LT.

Choledochocholedochostomy anastomosis and R-Y anastomosis are widely used in most transplantation centers. Choledochocholedochostomy anastomosis is frequently preferred because of the physiological continuity of the biliary system, technical advantages such as easier access to the biliary system after LT, prevention of bowel content reflux to the biliary duct, and shorter operative times.^7,8,14^ However, several studies have found that the incidence of biliary complications in recipients with CDCD anastomosis is higher than in recipients with R-Y anastomosis.^7,15-17^ Despite advancements in surgical techniques over the past 2 decades, biliary complications remain the most common problem following LT.^16-19^ A comparison of the rates in this study with those of our previous study^11^ showed that the incidence of biliary complications decreased from 36% to 31% over the last decade. However, the rate remains high compared to those reported in previous studies.^2,5,13^ This could be because we included only recipients undergoing CDCD anastomosis, and most patients were recipients of LDLTs. Previous studies have reported that LDLTs strongly correlate with biliary complications due to a small duct size, the presence of multiple duct orifices, and devascularization of the bile duct.^7,11,14,20^

Several risk factors including recipient and donor characteristics and perioperative and postoperative parameters are associated with the development of biliary complications following LT.^2, 7-10, 21^ However, the data are conflicting. Tingle et al^2^ found that graft donation after circulatory death, aberrant graft arterial anatomy, high recipient MELD scores, and long vascular anastomosis times were risk factors for early biliary complications following LT. However, in the present study, we found no significant association between recipients or donor factors and the development of biliary complications. Similarly, Jiménez-Romero et al^21^ identified no independent risk factors for posttransplant biliary complications.

Biliary complications after LT are also associated with patient and graft survival.^2,22^ A previous study reported that recipients with early biliary complications had significantly worse graft survival (84.5% vs. 75.1%, *P* < .001) and patient survival (83.3% vs. 76.9%; *P* < .001) rates than those without biliary complications,^2^ while there were no significant differences in graft survival between recipients who were successfully managed endoscopic and surgically.^2^ The early diagnosis and successful treatment of biliary complications after LT are crucial for preventing posttransplant morbidity and mortality. Currently, ERC is used as the first-line treatment for biliary complications.^5-7, 23^ The success rate (83%) of endoscopic interventions in the present study is consistent with previous reports.^2, 5, 11, 13, 20^ In the present study, 3 LDLT recipients with anastomotic strictures required revision surgery and 2 were successfully treated using the percutaneous rendezvous method. Six patients died due to biliary infection and sepsis.

In conclusion, early biliary stricture and leakages were the most common biliary complications after LT. Endoscopic treatment is successful in most recipients and contributes to better patient outcomes.

## Figures and Tables

**Table 1.** The Rate and Type of Biliary Complications After LT

Type of Biliary Complication	n (%)
Anastomotic stricture	29 (53.7%)
Anastomotic stricture + choledocholithiasis	10 (18.5%)
Biliary leak	7 (13%)
Biliary stricture + biliary leak	7 (13%)
Isolated choledocholithiasis	1 (1.8%)

**Table 2.** Treatment Modalities in Patients with Biliary Complications

Treatment Option	n (%)
Balloon dilatation + plastic stent placement	21 (38.8%)
Plastic stent placement	10 (18.5%)
Stone removal + plastic stent placement	6 (11.1%)
Isolated sphincterotomy	4 (7.4%)
Metallic stent placement	4 (7.4%)
Percutaneous rendezvous method	5 (9.3%)
Magnet method	1(1.9%)
Surgical revision	3 (5.6%)

**Table 3.** Factors Influenced on the Development of Biliary Complications Following LT

	Patients Without Biliary Complications (n = 120)	Patients With Biliary Complications (n = 54)	*P*
Age	51.2 ± 12.3	51.8 ± 12.0	.383
Gender (%) (female/male)	35%/65%	35%/65%	.136
Body mass index	26.8 ± 5.2	27.5 ± 4.9	.352
Pretransplant MELD score (median [min-max])	16 (6-40)	16 (6-41)	.94
Emergency transplant	6.7%	5.6%	.26
Deceased-donor transplantation (%)	14.2%	11.1%	.17
Living-donor lobe-segment (%) Right lobe/left lobe/right lateral segment	88.5%/10.5%/1%	85.7%/12.3%/2%	.08
The presence of HCC (%)	23%	19%	.126
On sirolimus or everolimus-based therapy (%)	29.1%	31.4%	.758

(Mean ± SD).

HCC, hepatocellular cancer; LT, liver transplantation; SD, standard deviation.
